# Hospital and Institutional News

**Published:** 1916-06-17

**Authors:** 


### HOSPITAL AND INSTITUTIONAL NEWS.

### OUR HOSPITAL SUNDAY NUMBER.

The Special Hospital Sunday Appeal Number of The Hospital is published this week in anticipation of Hospital Sunday on June 25, price 1d. The difficulties of the voluntary hospitals, administrative, professional, and financial, are not diminishing, and are not likely to diminish during the duration of the war; on the contrary, all their burdens are tending to increase, and in consequence it is more than ever important that there shall be no falling off in one of the most important sources of income of the hospitals of the London area—namely, the collection of the Metropolitan Hospital Sunday Fund. In this Special Number, besides the statistical returns of the year's work in the hospitals and medical charities of London, the Roll Call of the Sick, and the details of the special needs of many institutions, an illustrated account is given of the tomb of Rahere, founder of the premier British hospital (St. Bartholomew's), in the Church of St. Bartholomew the Great, Smithfield, and of the steps which have been taken to protect it against the possibility of Zeppelin attacks. A striking sermon lately preached in Guernsey, and an article on Shakespeare's medical allusions, are also included in the Special Number, which begins with a forcible article pointing out to the clergy and ministers of all denominations throughout London why Hospital Sunday this year is of such special importance to themselves as well as to the voluntary hospitals.

### TWENTY-FIVE YEARS AS HOSPITAL CHAIRMAN.

The members of the works committee of Wolverhampton and District Hospital for Women have presented an illuminated address to Mr. G. L. Gibbons, who has acted as chairman of the committtee of management for twenty-five years. Before attaining to this position Mr. Gibbons had been an active member of the committee, and has therefore watched the continued development of the hospital over a long period of years. Beginning as a dispensary for women in Cleveland Road, and limited to an out-patient department, there was some opposition when the proposal was first made to convert it into a special hospital. Mr. Gibbons, however, supported the new movement by arguments and donations, and eventually an institute definitely separated from the general hospital was decided on. A house in Chapel Ash was taken, and a sum of £15,000 not long afterwards had to be raised to build the present premises in Connaught Road, where Mr. Gibbons himself presented the site. The veteran chairman still looks ahead, and recently gave public expression to his own hope that the day may yet come when this hospital for women will be entirely free to any sufferer. The road to this is the creation of an endowment fund. Its achievement would be the best monument to Mr. Gibbons' chairmanship; and the fact that he is as much alive as ever to future needs should be an inspiration to all the devoted supporters of the institution.

### THE SOUTH LONDON HOSPITAL FOR WOMEN.

The announcement is made that this new hospital will be opened on July 4 by the Queen, and that Her Majesty will then receive purses for the support and upkeep of the institution. The buildings are on the south side of Clapham Common, facing that open space. The accommodation is designed for eighty patients, and includes four general wards, a children's ward, an isolation department, certain private wards, and *x*-ray and pathological departments. The private wards are arranged into single bedrooms at three guineas a week; rooms with two beds at two guineas; and cubicles in an eight-bed ward at one guinea. The isolation department is placed on the roof, where there is also a roof garden which can be transformed into an open-air ward commanding views of the Crystal Palace and other landmarks. Lady Cowdray is giving all the beds required for the hospital, and various associations of professional women are undertaking each to support the cost of a bed. We hope shortly to publish the plans and a full description of this hospital.

### AN ULSTER HOSPITAL AND INCORPORATION.

We have often discussed the advantages of incorporation from the standpoint of institutional managers. It is, therefore, interesting to see that the Ulster Hospital for Children and Women is the latest applicant for this privilege. For the sake of smaller institutions which may be looking ahead to the time when a charter of incorporation may seem to be desirable, we append the text of the two resolutions which have been passed by the general committee of the Ulster Children's Hospital. The preliminary steps are thus apparent—(1) "That this association be registered under the Companies Consolidation Act, 1908, as a company limited by guarantee, and not having a capital divided into shares; and that application be made to the Board of Trade for a licence directing the registration of this association with limited liability, without the addition of the word 'limited' to its name." (2) "That the board of management be, and the same is, hereby authorised to do all things necessary to obtain the incorporation of the association as aforesaid, and to settle and approve of a memorandum and articles of association, and to pay out of the funds of the association all costs and expenses properly incurred for such purposes."

### CHARITY ON THE SOUTH COAST.

The South and West Coast towns have been placed by circumstances in a far more favourable position than those resorts which are situated on the East Anglian coast line. Notwithstanding this, the Swanage Cottage Hospital has had to complain of insufficient local support, which, as a recent speaker put it, would probably not have been the case if the institution had been situated in France or Mesopotamia. Yet in so far as Swanage has avoided the financial difficulties of other coast towns nearer the theatre of war, so does its duty lie in maintaining its hospitals as a special thankoffering. When this has been done, its patriotic energies will still be free to support whatever branch of war hospital elsewhere may most appeal to the imagination of its population. A special committee to interest local support has been formed, and the first duty of this body must be to associate men and women in the district with the actual work of the institution. A hospital developing a series of subsidiary leagues and other bodies becomes a centre of interest, and their formation is the most practical method of arousing and maintaining local support.

### FEDERATED MALAY STATES HOSPITAL.

Thanks to the liberal financial support accorded by all classes of the community in the Federated Malay States, the committee of management of the military hospital established by these States at Blackmore End, Kimpton, Herts, has been able to increase the accommodation from 80 to 168 beds. Two new wards, each of 44 beds, have been erected in the grounds of the house kindly lent by the owner, Mrs. Vincent, the wards being similar in type to those of the Canadian Military Hospital at Orpington, Kent. The hospital is being equipped with an *x*-ray installation, the cost of which is being borne by Messrs. Edward Boustead and Co., of Leadenhall Street, E.C., in conjunction with Messrs. Boustead, Hampshire and Co., of the Federated Malay States. Captain G. D. Freer, R.A.M.C. (T.), formerly Principal Medical Officer, Selangor, is in charge of the hospital.

### NURSING TUBERCULOSIS IN HERTFORDSHIRE.

The progress of the work of prevention and treatment of tuberculosis in the county of Hertfordshire is most instructively detailed in Dr. Hyslop Thomson's recently published report. Circumstances have required the postponement of the erection of the proposed county sanatorium and hospital, but the dispensary system has been carried on at full work during the year, and its value as a centre round which the whole scheme revolves has been increasingly proved. To replace the lack of that accommodation for acute and advanced cases, which is essential if preventive warfare is to be carried out, the County Council is about to equip a suitable house to admit those for whom hospital treatment is most urgently required. The appointment of seven health visitors under the Maternity and Child Welfare Scheme of the County Council has enabled the dispensary nursing to be undertaken by these health visitors in the respective areas. The domiciliary attendance on insured patients has been continued without cost to the Insurance Committee, owing to the generous action of the County Nursing Association, and uninsured persons are looked after by the nurses of the district associations. It is obvious that health visitors and nurses must have an important influence in that home control which is so essential to the successful prevention of the spread of tuberculosis.

### FURTHER GIFTS TO CARDIFF HOSPITAL.

For the third week in succession there has been recorded the receipt of munificent gifts by King Edward VII.'s Hospital, Cardiff. In order to perpetuate the memory of Lord Kitchener locally, Sir William James Thomas, of Ynyshir, has contributed 1,000 guineas for the endowment of a bed in memory of the great soldier. The bed will be next to that endowed by Sir William last November in memory of Nurse Cavell. The second great gift that falls within the records of the week is also a memorial to a soldier. He was a young soldier, only twenty-three, but he lived long enough to be greatly trusted and to justify by his courage and devotion the confidence that was placed in him, and to win also the respect and affection of his fellow-officers and the men under his command. The gift was communicated by Mr. W. H. Seager, who has always shown a generous interest in the institution, in a letter from which we quote the following moving sentences:—"My wife and I will be glad to defray the cost of the new operating-theatre (two thousand five hundred pounds) in memory of our dearly loved son (2nd Lieut. W. H. Seager, 10th South Wales Borderers), who was killed in action at Neuve Chapelle, February 7, 1916, aged twenty-three. . . . We wish you and all those associated with you in this Christ-like work all joy and happiness, and pray that success will crown all your labour."

### INSTITUTIONAL ENDOWMENTS OCCASIONED THROUGH THE WAR.

The recent losses in the great naval battle in the North Sea have resulted in an increase in the demands made upon the accommodation of the Port of Hull Society Sailors' Orphan Homes; and it is announced that Mr. T. R. Ferens, M.P., and Mrs. Ferens have given 2,000 guineas towards the proposed extensions which are in contemplation at this institution. Another institution has also received an addition to its endowments through the results of the war: Mr. Robert Cooper, of Bexley, has given £1,000 in Exchequer Bonds to the Bexley Cottage Hospital Endowment Fund as a memorial to his son, the late Lieutenant Sydney Gordon Cooper, who died of wounds in France. We can conceive no more excellent memorial of our gallant sailors and soldiers than the endowment of beds or wards in hospitals; and we are very pleased to see that bereaved parents and relatives are in so many cases acting upon this suggestion, which has been made already in these columns.

### KING'S COLLEGE HOSPITAL.

From offices at 97 Cannon Street a Special Appeal Committee is endeavouring to raise at least £100,000 for this institution, two-thirds of which sum is required to pay off actual debt on the rebuilt hospital and the remainder for carrying on the work of the institution. The chairman of the Appeal Committee is Mr. L. A. Martin, and the pamphlet which sets forth the needs of "King's" is admirably brief, interesting, and to the point. Not many months ago we had occasion to comment on the frank and courageous way in which the management of this hospital admitted their financial difficulties, and it is clear that those difficulties are being boldly faced and tackled. We are convinced that this is always the right course for a voluntary hospital to pursue, and we expect to see Lord Hambleden, Mr. Martin, and their colleagues on the committee of management well supported by the public in response to the present appeal.

### EXTENSION OF QUEEN MARGARET COLLEGE.

In consequence of the greatly increased enrolments of women medical students at Glasgow this year, it was reported at a meeting of Glasgow University Court on June 8 that additional accommodation is required for the instruction of women students. The Principal, Sir Donald MacAlister, asked the Court for authority to make some additions to the equipment of Queen Margaret College to meet this need. All existing accommodation, he stated, in the departments of Anatomy and Chemistry is overflowed; and the number of medical students had doubled during the year. No estimate of the cost of the necessary alterations and rearrangements was submitted, but the Principal urged that the situation must be faced and dealt with, and the Court took the same view by sanctioning the proposal, which gives Sir Donald practically a free hand to do what he finds necessary.

### THE CAVENDISH LECTURE.

The subject of the Cavendish Lecture to be delivered by Sir John Bland-Sutton, F.R.C.S., on June 23, at the West London Hospital, is "The Fate of Patients who have had Stones removed from the Kidney." Since the needs of the Army became so insistent there has been but little time for original work, especially on the part of surgeons, except upon problems directly concerned with military surgery. It is therefore all the more gratifying to find that so eminently practical and important a topic has been chosen for this lecture, and one upon which so little has been published since the great rise and progress of renal surgery which has taken place in the last few years. Sir John Bland-Sutton is an officer on the staff of the 3rd London General Hospital, so that the preparation of his address must have entailed considerable inroads upon the scanty leisure which this eminent surgeon is able to enjoy after his institutional day's work is done and his private patients have been attended.

### THE B.M.A. AND THE REMUNERATION OF POOR-LAW MEDICAL OFFICERS.

The local branch of the British Medical Association at Wigan has acted with commendable promptitude and force in dealing with the appointment of a temporary medical officer to the Wigan Poor-Law Infirmary. Dr. Litherland had offered to take the post at a salary of £200 per annum and extra fees. The Association at once called a meeting and asked Dr. Litherland to attend. It was pointed out that Dr. Litherland was taking the post and assuming the responsibility of a full-time and principal medical officer. In these circumstances the Association considered his salary should be at least £400 or £450 per annum, with extra fees, and that his offer was unfair to his brother practitioners. The arguments used convinced Dr. Litherland, and he resigned his appointment. Both the Association and Dr. Litherland are to be congratulated upon their action. If their example were followed in other Unions the under-payment of Poor-Law medical officers would soon be a thing of the past.

### IN PLACE OF THE ANNUAL DINNER.

When we say that times change, we mean, if we are men of business, that a dress is required for an old custom, and the art of maintaining income in times of change is largely the art of adapting old principles by giving them another name. One of the old-established principles of income-raising for hospitals has been the annual festival dinner or banquet. These it has been felt untimely to hold during the war; and hospitals which made a habit of relying on them for a large sum of money annually have been forced to seek a substitute. One of these, amusingly enough, is the festival luncheon. "Luncheon" sounds less elaborate and costly, but as frequenters of fashionable hotels know to their cost, the number of courses served at 1 P.M. is scarcely smaller than the number presented at 8. We observe that some luncheons have been successfully held of late, and commend this success to hospital officers fearing that they must abandon, or actually having cancelled, this function. A city hall, if procurable, or some place with a *cachet* of its own, is better than any hotel, however expensive.

### IS A SEPARATE CHAPEL ESSENTIAL FOR WORSHIP?

Denominational differences, happily, have not threatened the Halifax Guardians' acceptance of a hut for religious services offered to them by Archdeacon Norris for the benefit of patients in the War Hospital associated with the board. The committee resolved to accept the offer on the understanding that once in each Sunday, and one other evening during the week, services could be held there by Nonconformist bodies. This was agreed to, but the Guardians did not bind themselves to use the building solely for religious purposes after the war. On behalf of Archdeacon Norris, it was suggested that the main object of the gift was to secure a place wholly devoted to religious observances, since the previous use of the Y.M.C.A. hut for these purposes had not been found conducive to worship. Medical superintendents of Poor-Law infirmaries, where there is not always a chapel for the staff, can easily be found to support this view, and the practical objection to using a board-room for Sunday services rests upon the observation that a place of worship should have its own associations and its own atmosphere.

### THE COMPULSORY REMOVAL OF PHTHISICAL PATIENTS.

The recent discussion at a meeting of the West Riding Insurance Committee upon the question of the enforced segregation of consumptive patients under certain conditions raises the whole point of infectivity in tuberculosis. Speaking generally, it may be said that the average case of phthisis in a sanatorium or hospital, or even under private medical care, is not a source of danger to the community at large. The "after-care" patient, upon his discharge from a sanatorium, has been too well drilled in the rules of personal hygiene for him to indulge in the habit of promiscuous expectoration. The cases that may be considered as really infectious from the public health standpoint are those of advanced pulmonary disease, or those with discharging sinuses in connection with viscera, bones, or joints, when their home surroundings are unfavourable. It stands to reason that the health of "contacts" is likely to be gravely prejudiced when two or three persons share the same ill-ventilated room with a patient suffering from tuberculosis of the "open" type. Public sentiment, however, appears to be hardly advanced enough to adopt the principle of compulsory removal of a consumptive, even in the interests of public health, without a good deal of opposition. At present public health authorities have no power to enforce isolation or removal, even in cases where the risk of infection from a tuberculous patient is obviously great.

### COAL-DUST AND PHTHISIS.

Those persons who have been unduly pessimistic with regard to the health of coal-miners should be relieved by Dr. J. S. Haldane's pronouncement that the death-rate from phthisis among colliers is lower than in nearly all other occupations. Speaking at the annual meeting of the Institution of Mining Engineers last week, he dwelt upon the fact that coal-dust in the lung appears to afford some protection against the onslaught of tuberculosis. In a well-marked case of anthracosis the lungs are black and emphysematous, and a large amount of carbonaceous pigment may be demonstrated *post mortem* as well as the presence of small black nodules. Coal-dust cannot be called an antiseptic, so that the practical immunity enjoyed by miners against phthisis must be explained upon other grounds. The theory advanced by Dr. Haldane that the pulmonary epithelium is educated to deal with harmful foreign bodies by the constant introduction of smoky particles into the lungs, may afford comfort to dwellers in sooty atmospheres. In moderation, therefore, coal-dust may be regarded as a safeguard to the health of colliers, apart from the risk of explosions.

### A HOLIDAY HOME AS A SCHOOL.

The growth of special schools for children whose physique or temperament requires that expert attention which the elementary school teacher has neither the opportunity nor the training to supply is, on the whole, a welcome one. The recent Acts empowering education authorities to provide these schools are, in fact, a necessary corollary of our national system of education, just as were medical inspection and free meals. Oldham has taken advantage of the powers at its disposal to meet the needs of various classes of children defective either in body or in mind. The latest departure in this line is the sanatorium school at Castleshaw, formerly, and we hope faithfully, known as Holiday Home. It is important that the technical school which replaces it, whether or not it keeps the old name, should live valiantly up to the old title. To avoid misconception, let us add that one of the scientific names for the process of making work and play regarded as interchangeable terms from the child's point of view (it makes the highest demands upon the teacher) is the Montessori system. The new Oldham Sanatorium School, finely started with such a tradition as the old name of Holiday Home provides, should fill not only the lungs of the children with fresh air, but their souls with the spiritual oxygen of freedom.

### THE REFORM OF THE FUNERAL.

Probably the one trade paper which is never likely to have an enormous circulation among the laity is the *Undertakers' Journal*, which ventilates the hopes and fears of those engaged in a very important business associated with the public health. None the less, the public as well as the undertakers are almost equally concerned in the new scheme for funeral reform which has been proposed, and, in some sort, recognised in Liverpool. Mr. J. G. Taggart, chairman of the Liverpool Burials Committee, under which the six public cemeteries of the city are now administered, is suggesting to the City Council a comprehensive scheme of funeral reform. In the belief, supported by those likely to know, that £10 is the least sum spent on funerals by all above the actual poverty level, Mr. Taggart suggests the economy that would be effected if all funerals could be undertaken by the municipality. His scheme comprises compulsory notification of death, wherever it occurs, at the Health Office; the attendance of the medical officer to certify death, to obtain any publicly important information from the practitioner in attendance, to remit to the Coroner any facts which he should know, and to give such directions as the interests of the living might require. On his heels, so to speak, an electrically propelled hearse would follow, to remove the body to a mortuary chapel at one of the cemeteries; and by this means the reverent and speedy removal of the body, always important where house-room is small, would be secured. The scheme further aims at curtailing the opportunities for the display of "a cortège" with its crowd of sightseers; at the avoidance of ostentatious obsequies; and at consequent reduction of large funeral expenses on the part of the poor. These things, however, are popular; but Mr. Taggart hopes that the suggestion of state afforded by the municipal hearse would provide all that the most ardent mourner could desire. The Sunday funeral has ceased. Will the municipal funeral succeed it?

### SCOTLAND AND MENTALLY INCAPACITATED SOLDIERS.

The question of the treatment of soldiers mentally incapacitated has been taken up by a combination of Edinburgh, Glasgow, and Govan Parish Councils, who have issued a memorandum setting forth their views. It is urged that such patients ought not to be relegated to a district asylum and treated as poor persons chargeable to the local rates, but that the burden should be undertaken by the National Exchequer, and in view of the temporary character of the existing arrangements for the reception of mental cases in military hospitals, permanent accommodation should be provided by the Army authorities in separate institutions set apart for the specific purpose by the Government. It is likely that the Scottish authorities named will take further steps to bring their views before the Government Departments concerned.

### HOSPITAL INVESTMENTS IN WAR LOAN.

The managers of the Forres Leanchoil Hospital have announced that the £10,000 legacy received from the late Lord Strathcona has been invested as follows: The sum of £5,000 in Exchequer Bonds at 5 per cent., and the remaining moiety in one local and one Edinburgh mortgage security. We need not remind our readers that the above forms one of the largest legacies ever received by a small hospital, and that Lord Strathcona had long been closely associated with it.

### "BLIGHTY: THE FREE PAPER."

The scarcity of paper has affected not only readers at home: sailors and soldiers no longer get the unsold copies returned to the offices from the newsvendors. Returns exist no more. This fact, we learn, led a father of two sons, in the Navy and Army respectively, to consider the plan of making a literary digest of pictures, stories, and jokes, so as to form a newspaper, and to circulate it free to men in the Services. The newspaper proprietors' only condition apparently was that no copies of the new journal should be sold at home. A public subscription was started. The War Office, the Y.M.C.A., the Red Cross Society, the Navy League, and the British and Foreign Sailors' Society agreed respectively to act as distributing centres. Its object is to make the characteristic yarns of either Service current in the other, but until a plentiful supply of these is obtained from readers, a variety of other newspapers is drawn upon to provide the series of "comic" drawings, stories, and tales of which *Blighty* (from *Bilati*, an Eastern word corrupted by soldiers and applied to Great Britain) is at present composed. It has been the experience of the most successful war hospital gazettes that something richer than quips and merely funny "stories" makes in the long run the better reading. *Blighty* will probably find out the same truth.

### THE MAY "GAZETTES" FROM CAMBRIDGE.

One of the features of the 1st *Eastern General Hospital Gazette* is its custom of differently coloured covers; but it also appears to find more difficulty than its contemporaries in procuring a decent supply of paper. The transparency of that used in last month's issue, for instance, is very disagreeable, and it does not do justice to the blocks. There is a good supply of funny verse, among which, a somewhat unusual feature, a nurse lets herself go in regard to her patients in the same way as sentimental patients often do in regard to their nurses. The "stories" have the merit of being apparently the result of genuine hearsay and observation, and not the product of a contributor whose melancholy task it is to manufacture them for publication. In the "Rules for Officer Patients" there is a pleasant note of satire; but there is a blunt frankness about rule 8, which reads: "Get out as soon as you can—nobody really wants you here." We note that women clerks have lately been employed in the hospital office, and that reports are favourable concerning their efficiency. There is an able skit on the economy campaign in this number, which is all the better reading for the care with which it has been drawn up.

### THE R.A.M.C. DEPOT MAGAZINE.

The bulky and beautifully produced *Journal* of the Royal Army Medical Corps is admittedly in no sense a paper for the rank and file; it is a scientific monthly of high standing and great excellence. Possibly the success of many of the topical and popular magazines run by various general hospitals has caused the non-commissioned ranks of the R.A.M.C. to realise as never before that they possess no paper of their own. At any rate, this omission is now rectified by the publication of a paper bearing the above title, edited by a private and a corporal of the Corps, and published at one penny. Until the magazine gets well into its stride rigorous criticism would be out of place: suffice it that the editors have undoubtedly unearthed a capable caricaturist. We shall watch this venture with especial interest, because obviously it is one which is not predestined from the nature of things to perish when peace is declared, but may quite possibly establish itself as a permanency.

### CORONERS' JURIES IN WAR TIME.

A letter in the *Daily Mail* suggests that at a time when the scarcity of labour is so great that it may involve the stoppage of many of the amenities of civilised life which are no longer luxuries, "hundreds of able-bodied men waste their time every day on Coroners' juries." The writer adds, quite truly, that a large number of the cases are purely formal inquiries, at which the medical evidence clinches the whole matter and is instantly accepted by Coroner and jury. The mere non-attendance of a medical man in *prima-facie* cases of sudden death should not, says the *Daily Mail's* correspondent, necessitate the calling of a jury when the *post-mortem* examination shows the death to have been natural. All this is very true, and holds good for peace time as well as war. Reform of the Coroner's Court has been long overdue, but amid the clash of party warfare it has no chance of obtaining any attention from parliamentarians, because there are no votes to be carried by adding it to the party platform. Perhaps the war, by stilling party faction and by drawing attention to the sources of waste in our national economy, may make also for the much-needed reforms which are widely admitted by competent authorities to be desirable in the procedure of Coroners' inquests.

### THE NEW SECRETARY OF THE ROYAL HAMPSHIRE HOSPITAL.

We have had to hold over till this week news of the appointment of Mr. E. I. Pearce to the secretaryship of the Royal Hampshire County Hospital. It is interesting to note that Mr. Pearce has proceeded by way of a private secretaryship to that of a public institution. After some training in a business firm he became secretary to Lord Derwent, of Scarborough. So far as our information goes this is his first direct experience of hospital work. There were 120 candidates for the post, a vacancy in which was lately created by the death of Mr. A. D. White, who had held the office of secretary for five years. Mr. White's death was a shock to the hospital and his friends, and the committee passed a special resolution expressing their own "deep sorrow" and their recognition of his zeal and ability.

### THE WAR STANDARD OF SANITATION.

There is real practical work to be done at the conference on sanitary administration under war conditions, which, according to information from Leeds, is about to be held at the Royal Sanitary Institute. The fact is that the municipal standard, which the public interest demands should be kept as high and exemplary as possible, has been threatened here and there by ill-considered proposals and propaganda in the name of "national economy." Our noodles are apt to regard careful watering of the streets in dry weather as wasteful, and the no less careful and regular collection of house rubbish as wasteful, until the inevitable epidemic which comes on the heels of neglect in these directions brings, not the agitators, but the ratepayers face to face with heavy and avoidable expenditure. It is therefore good to learn that Dr. James Wheatley, medical officer of health for Shropshire, and other speakers, are to deal with the maintenance of the municipal standard; and we hope that they will make the fullest use of the occasion to drive well home the economy of preventive measures.

### A CONTUMACIOUS COUNCIL.

The Local Government Board is insisting that local authorities shall take measures to safeguard infant life. So far the Islington Borough Council has persistently refused to do anything despite a report of the Medical Officer of Health urging the need of an infant welfare scheme. Now the Local Government Board has sent the Borough Council a letter stating that the President of the Board regrets that the Islington Borough Council have not yet taken steps to provide for the systematic visitation of infants whose births are notified in the borough and who require visiting; that among the 111 Metropolitan and County Boroughs in England and Wales there are less than ten in which the local authority has not appointed a staff of health visitors, and of these Islington is by far the largest in population and one in which, owing to the circumstances of the district, this work is especially needed; that the Borough Council is incurring a grave responsibility in continuing to delay the initiation of this work, particularly at a time when the importance of conserving infant life is paramount; and that Mr. Long hopes that the Council will at an early meeting decide to appoint the five health visitors recommended in the Medical Officer of Health's report, in addition to the two sanctioned by the Council for the purpose of visiting cases of measles and other infectious diseases.

### THIS WEEK'S DRUG MARKET.

The downward tendency in the prices of several important drugs continues; this is especially noticeable in the case of bromides and the salicylates. For some time past the market for bromides has been quiet, and the demand was not greatly stimulated when a short time ago makers announced a substantial reduction in their quotations, and even now when the makers are apparently willing to sell at something below their nominal quotations business is by no means brisk. Salicylic acid and salicylate of soda are appreciably lower in price than they were a short time ago, and the steady downward tendency continues; this is mainly due to the fact that both English and American makers are producing in larger quantities. Acetanilide, phenazine, and sulphonal are also slightly lower in price, and the advanced value of phenacetin is not quite so firmly maintained as it was. Bismuth preparations have a slightly lower price tendency. Citric acid and tartaric acid are about unchanged, but lower values may perhaps be expected in the near future. Potassium permanganate is again dearer and supplies are scarce. There has been a good demand for cocaine, and the lower tendency in price which had been noticeable during the past few weeks has for the time being ceased.

### TO OUR READERS.

Contributions are specially invited from any of our readers to these columns. **They should deal with topical subjects and news. They must be authenticated for the information of the Editor only. The minimum payment if published is 5s. There is no hard-and-fast rule as to space, but** notes of about twenty lines in length are preferred.

